# Clinical Characteristics and Prognosis of Idiopathic Acute Pancreatitis

**DOI:** 10.5041/RMMJ.10442

**Published:** 2021-07-20

**Authors:** Tzvika Porges, Tali Shafat, Iftach Sagy, Dan Schwarzfuchs, Ilan Rahmani Tzvi-Ran, Alan Jotkowitz, Leonid Barski

**Affiliations:** 1 Internal Medicine Division F, Soroka University Medical Center, Beer-Sheva, Israel; 2 Clinical Research Center, Soroka University Medical Center, Beer-Sheva, Israel; 3 Rheumatologic Unit, Soroka University Medical Center, Beer-Sheva, Israel; 4 Department of Emergency Medicine, Soroka University Medical Center, Beer-Sheva, Israel; 5 Faculty of Health Sciences, Ben-Gurion University of the Negev, Beer-Sheva, Israel

**Keywords:** Acute pancreatitis, drug induced pancreatitis, idiopathic pancreatitis

## Abstract

**Objective:**

Acute pancreatitis is a serious diagnosis with an increasing incidence in the Western world. In this study we sought to investigate the incidence of idiopathic AP and to compare clinical and prognostic characteristics of idiopathic cases with cases of AP with known etiology.

**Methods:**

In this retrospective study of adult hospitalized patients diagnosed with acute pancreatitis between 2012 and 2015, a comparison was made between admissions of patients with known etiology and those for whom no cause was found. Primary outcome was defined as composite outcome of 30-day mortality and complications.

**Results:**

Among 560 admissions of 437 patients with a primary diagnosis of acute pancreatitis, the main factors identified were gallstones (51.2%) and idiopathic pancreatitis (35.9%), with alcohol ranked third at only 4.8%. Mortality rate within 30 days of hospitalization was 2.9% and within one year was 7.1%. Use of lipid-lowering, anti-hypertensive, and anti-diabetic medications was more frequent among patients with “idiopathic” disease (70%, 68%, and 33% versus 59%, 56%, and 27%, respectively). Patients admitted with idiopathic AP, in comparison to patients with known AP etiology, had milder disease with shorter hospital stay (3 days versus 4, respectively), and less re-admission in 30 days (7.5% versus 21.2%). Idiopathic AP patients had better prognosis in terms of 30-day death and complication (HR 0.33, 95% CI 0.08–0.40, *P*<0.001).

**Conclusion:**

Idiopathic disease is common among acute pancreatitis patients; the two study groups differed in severity of disease and prognosis. Common use of medications with doubtful value suggests possible under-diagnosis of drug-induced acute idiopathic pancreatitis.

## INTRODUCTION

Acute pancreatitis (AP) is an acute inflammatory process of the pancreas. Annual incidence of AP is in the range of 4.9–35 cases per 100,000 people, with a mortality rate of between 5% and 10% of hospitalized patients.^1^–^3^ In the past few decades there has been an increase of incidence, with a decline in mortality.^4^ These trends are probably due to increased prevalence of obesity and alcohol consumption, along with better diagnostic methods and improved medical care.^3^ It is known that the two most common etiologies for AP are obstructive gallbladder disease (up to 40% of AP cases) and alcohol use (up to 30% of AP cases).^5^,^6^ Additional but less common etiological factors include hypertriglyceridemia, hypercalcemia, and resulting from endoscopic retrograde cholangiopancreatography (ERCP) and drug reaction, among others.

In the absence of etiology it is possible to perform ERCP including aspiration and microscopic examination of the gall contents, magnetic resonance cholangiopancreatography (MRCP), and high-resolution endoscopic ultrasound (EUS). These methods can help to diagnose microlithiasis and other possible factors causing AP.^7^,^8^ Extensive investigation including advanced imaging techniques such as MRCP, ERCP, EUS, manometry, and gall analysis according to the American Gastroenterological Association (AGA) guidelines still leaves 15%–25% of cases as idiopathic.^5^

In a study from Australia published in 2015, the percentage of AP patients with no identified cause was 25.9%; the authors found no significant prognostic difference between these patients and patients with known etiology.^9^

In this study we sought to estimate the prevalence of idiopathic AP and to compare clinical and prognostic characteristics of patients hospitalized with an AP diagnosis with known etiology versus idiopathic AP. Furthermore, we intended to follow the group of idiopathic AP patients for a period of one year, to investigate whether etiology for their disease was eventually determined.

## METHODS

### Study Population

This retrospective cohort study included patients aged 18 and above who had been admitted with a primary diagnosis of acute pancreatitis (ICD-9 code 577.0) in Soroka University Medical Center (SUMC), a tertiary hospital that serves one million residents of the southern region of Israel, during the years 2012–2015.

For each admission, demographic and clinical data were collected, including background diseases, chronic medication use, admission diagnosis, hospitalization vitals, and laboratory and imaging results. Two internal medicine physicians reviewed patients’ files, and for each case the probable etiology of AP was determined. Cases with no known etiology were defined as idiopathic.

### Outcome Measures

Primary outcome was defined as composite outcome including 30-day mortality, intensive care unit (ICU) admission, complications of pancreatitis (e.g. necrotizing pancreatitis, pseudocyst), surgery due to complications, and 30-day re-admission due to pancreatitis (omitting ambulatory admissions for ERCP or surgery). Secondary outcome was all components of the composite outcome, and long-term (1 year) outcome.

### Follow-up

“Idiopathic” pancreatitis patients’ medical records were examined for a period of 12 months after discharge in order to ascertain AP etiology in retrospect. During that year, outpatient clinic, imaging, and hospital re-admissions were reviewed.

### Statistical Analysis

Data are expressed as mean±standard deviation (SD), median±interquartile range (IQR), or number and percentage. We compared patient characteristics between idiopathic versus “non-idiopathic” pancreatitis using *t* test, chi-square test, and non-parametric test. We used generalized estimating equation (GEE) logistic regression (unstructured matrix) to compare proportions of composite outcome between the two groups. This accounts for the clustering of the same patient composite outcome risk. The final model was selected based on model goodness-of-fit, minimal covariates, and plausible clinical explanation. Statistical analysis was performed using the Statistical Package for Social Sciences for Windows (SPSS), version 25.0 (IBM Corp, Armonk, NY, USA).

The study was approved by the Institutional Review Board prior to its initiation. Confidentiality was maintained throughout the study.

## RESULTS

### Baseline Patient Characteristics

Between the years 2012 and 2015, there were 560 admissions of 437 patients enrolled in the Israeli health maintenance organization, Clalit Health Services, aged 18 and above to SUMC with a primary diagnosis of AP.

**Figure 1:**
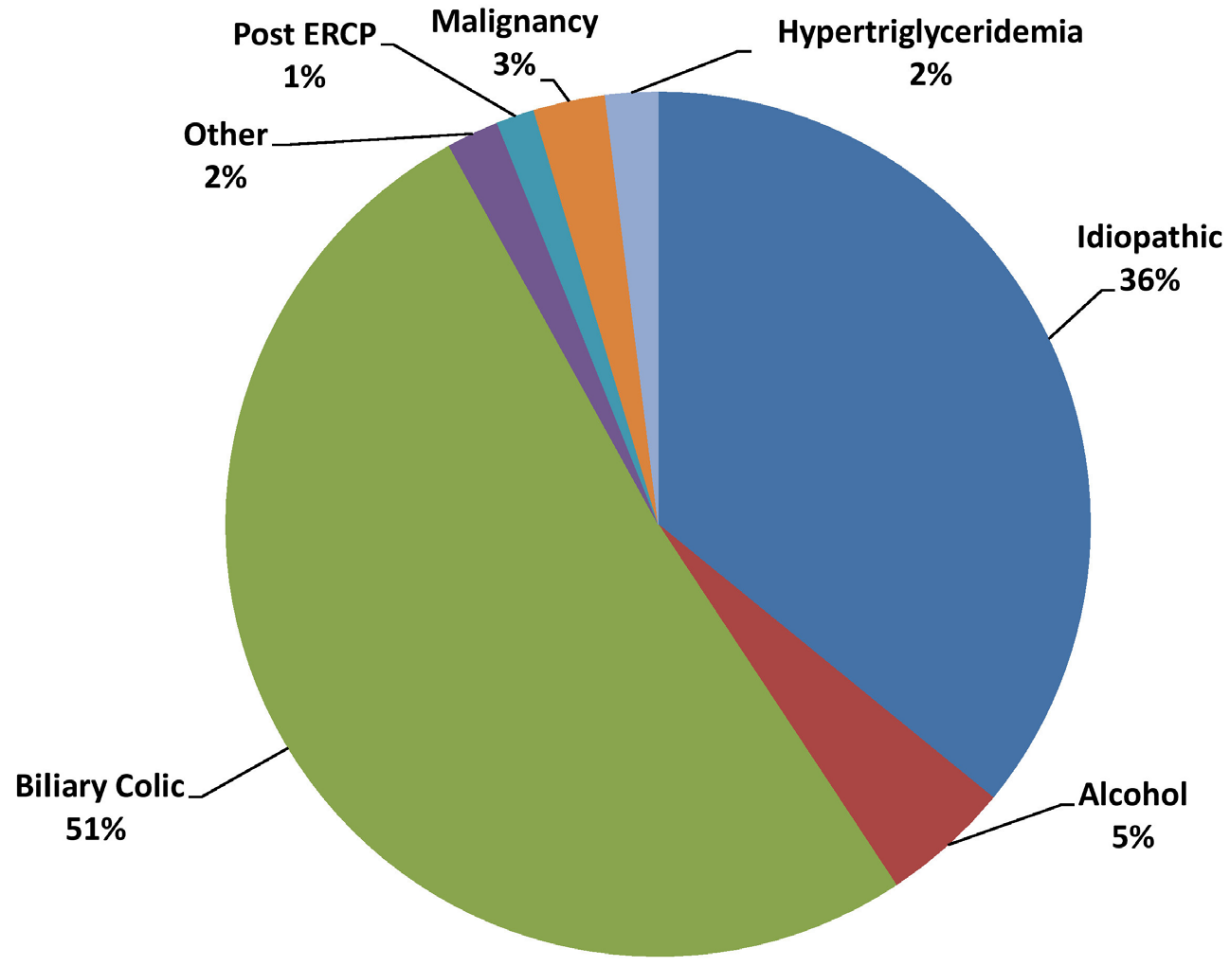
Distribution of Probable Acute Pancreatitis Etiology at Hospital Discharge. ERCP, endoscopic retrograde cholangiopancreatography.

The main probable etiologies were gallstones (51.2%) and idiopathic AP (35.9%), with alcohol in third place at only 4.8% of cases (Figure 1). Mean age was 61.9 years, 46.3% were male, and 86% were Jewish. The proportion of patients with Charlson co-morbidity index >1 was 17.7%. Other background diseases are listed in Table 1. According to the modified Glasgow score, 18.4% of cases were classified as severe, and 3.6% suffered complications, the most common of which was necrotizing pancreatitis (2.7%). Of all AP admissions, 2.1% eventually transferred to the intensive care unit.

**Table 1:** Background Characteristics and Clinical Data of Study Population According to Acute Pancreatitis with Identified versus Unknown Etiology.

Variable	Total (*n*=560)	Identified Etiology (*n*=359)	Idiopathic (*n*=201)	*P* Value
**Demographics**				
Males, *n* (%)	259 (46.3)	153 (42.6)	106 (52.7)	0.022
Age, years, mean±SD	61.9 (19.1)	62.5 (18.9)	61.0 (19.5)	0.376
Jewish, *n* (%)	483 (86.3)	307 (85.5)	176 (87.6)	0.525
Israel born, *n* (%)	217 (38.8)	133 (37.0)	84 (41.8)	0.279
**Background Diseases**				
Chronic ischemic heart disease, *n* (%)	37 (6.6)	26 (7.2)	11 (5.5)	0.481
Dyslipidemia, *n* (%)	180 (32.1)	121 (35.1)	59 (32.4)	0.563
Chronic obstructive pulmonary disease, *n* (%)	41 (7.3)	29 (8.1)	12 (6.0)	0.402
Diabetes, *n* (%)	159 (28.4)	92 (25.6)	67 (33.3)	0.063
Chronic kidney disease, *n* (%)	72 (12.9)	45 (12.5)	27 (13.4)	0.793
Liver disease, *n* (%)	124 (22.1)	88 (25.5)	36 (19.8)	0.161
Malignancy, *n* (%)	34 (6.1)	26 (7.5)	8 (4.4)	0.194
Charlson co-morbidity index >1, *n* (%)	99 (17.7)	61 (17.0)	38 (18.9)	0.566
Previous admission with pancreatitis, *n* (%)	123 (22.0)	83 (23.1)	40 (19.9)	0.397
**Chronic Medications**				
ACE/ARBs, *n* (%)	247 (44.1)	149 (56.0)	98 (68.1)	0.020
Diuretics, *n* (%)	88 (15.7)	58 (21.8)	30 (20.8)	0.900
Anti-diabetic drug, *n* (%)	93 (16.6)	46 (17.3)	47 (32.6)	0.001
Lipid-lowering drug, *n* (%)	258 (46.1)	157 (59.0)	101 (70.1)	0.032
**Vital signs on admission**				
O_2_ saturation <90%, *n* (%)	7 (1.3)	1 (0.3)	6 (3.0)	0.006
**Modified Glasgow Score**				
Albumin <3.2 g/dL, *n* (%)	83 (14.8)	65 (18.1)	18 (9.0)	0.004
Calcium <8.0 mg/dL, *n* (%)	34 (6.1)	27 (7.5)	7 (3.5)	0.065
Glucose >180 mg/dL, *n* (%)	66 (11.8)	41 (11.4)	25 (12.4)	0.785
LDH >600 U/L, *n* (%)	74 (13.2)	54 (15.00)	20 (10.0)	0.092
Urea >45 mg/dL, *n* (%)	125 (22.3)	82 (22.8)	43 (21.4)	0.751
WBC >15.0×10^3^/μL, *n* (%)	74 (13.2)	51 (14.2)	23 (11.4)	0.435
ALT >150 U/L, *n* (%)	174 (31.1)	135 (37.6%)	39 (19.4)	<0.001
Modified Glasgow score >2, *n* (%)	103 (18.4)	71 (19.8)	32 (15.9)	0.306
Lipase, U/L, mean±SD	737±1129	850±1186	615±1012	0.061
Amylase, U/L, mean±SD	451±534	498±561	367±469	0.004

ACE, angiotensin-converting enzyme; ALT, alanine aminotransferase; ARBs, angiotensin receptor blockers; LDH, lactate dehydrogenase; WBC, white blood cell.

### Comparison between “Idiopathic” AP and “Known Etiology” AP

In the idiopathic patients group, the proportion of male patients was significantly higher (52.7% versus 42.6%, *P*=0.022), as was the trend of a background diagnosis of diabetes mellitus (33.3% versus 25.6% *P*=0.063). Patients without known etiology were more prone to use medications that interfere with the renin–angiotensin–aldosterone system, as well as anti-diabetic and lipid-lowering medications compared to patients with known etiology.

### Outcomes

Patients with idiopathic disease, as compared to non-idiopathic AP, had milder disease as reflected by shorter hospital length of stay (3 versus 4 days, *P*<0.001) and decrease of both short (30-day) and long-term (1-year) re-admission rates (7.5% versus 21.2%, *P*<0.001; and 28.3% versus 46.5%, *P*<0.001, respectively) (Table 2).

**Table 2:** Prognostic Characteristics According to Acute Pancreatitis with Identified versus Unknown Etiology.

Characteristic	Total (*n*=560)	Identified Etiology (*n*=359)	Idiopathic (*n*=201)	*P* Value
**Short-term Outcomes**				

Admitted to ICU, *n* (%)	12 (2.1)	9 (2.5)	3 (1.5)	0.426

Length of hospital stay, days, median (range)	4.0 (2.0–6.0)	4.0 (3.0–6.0)	3.0 (2.0–5.0)	<0.001

Any complication, *n (*%), of which:	20 (3.6)	14 (3.9)	6 (3.0)	0.643
Necrotizing, *n* (%)	15 (2.7)	10 (2.8)	5 (2.5)	0.740
Pseudocyst, *n* (%)	5 (0.9)	4 (1.1)	1 (0.5)	

Surgery due to complication, *n* (%)	1 (0.5)	3 (0.8)	0 (0.0)	0.194

30-day re-admission due to pancreatitis, *n* (%)	54 (9.6)	49 (13.6)	5 (2.5)	<0.001

30-day re-admission due to any cause, *n* (%)	91 (16.3)	76 (21.2)	15 (7.5)	<0.001

30-day ERCP/MRCP tests, *n* (%)	29 (5.2)	26 (7.2)	3 (1.5)	0.002

30-day mortality, *n* (%)	16 (2.9)	11 (3.1)	5 (2.5)	0.769

Composite outcome, *n* (%)*	86 (15.4)	70 (19.5)	16 (8.0)	<0.001

**Long-term Outcomes**				

365-day mortality, *n* (%)	40 (7.1)	25 (7.0)	15 (7.5)	0.865

365-day re-admission due to pancreatitis, *n* (%)	112 (20.0)	92 (25.6)	20 (10.0)	<0.001

365-day re-admission due to any cause, *n* (%)	224 (40.0)	167 (46.5)	57 (28.4)	<0.001

365-day ERCP/MRCP tests, *n* (%)	78 (13.9)	52 (14.5)	26 (12.9)	0.703

Gastro outpatient clinic follow-up, *n* (%)	147 (26.3)	80 (22.3)	67 (33.3)	0.005

* Composite outcome composed of 30-day mortality, ICU admission, any complication, surgery due to complication, and 30-days re-admission due to pancreatitis.

ERCP, endoscopic retrograde cholangiopancreatography; ICU, intensive care unit; MRCP, magnetic resonance cholangiopancreatography.

**Figure 2:**
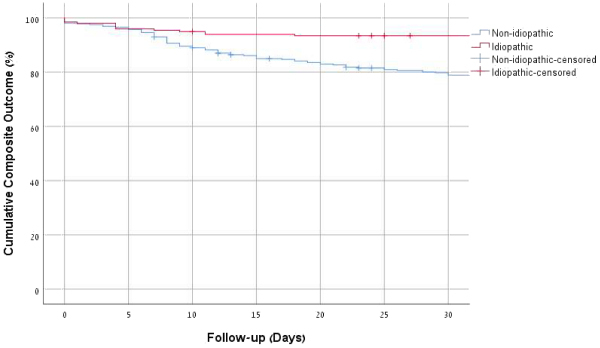
Kaplan–Meier Survival Curve of Composite Outcome (30-day Mortality, ICU Admission, Any Complication, Surgery Due to Complication, and 30-day Re-admission Due to Pancreatitis). Log rank *P*=0.001.

In univariate analysis (Kaplan–Meier curve), idiopathic disease had better outcome according to significant difference in composite outcome (log rank *P*<0.001) (Figure 2).

In multivariate analysis, idiopathic disease was associated with better prognosis according to composite outcome (including 30-day mortality, ICU admission, any complication, surgery due to complication, and 30-day re-admission due to pancreatitis) (HR 0.33, 95% CI 0.08–0.40, *P*<0.001, adjusted to Glasgow score, gender, Charlson index, and lipid-lowering treatment) (Table 3). Cox regression analysis shows that in addition to having unknown etiology (idiopathic disease), positive endpoints were also significantly more common among patients who used lipid-lowering drugs and had a lower modified Glasgow score (Table 3).

**Table 3:** Multivariate Analysis (Generalized Estimating Model) of Composite Outcome (30-day Mortality, ICU Admission, Any Complication, Surgery Due to Complication, and 30-day Re-admission Due to Pancreatitis).

Parameter	HR	95% CI	*P* Value
Idiopathic disease	0.33	0.08–0.40	<0.001
Glasgow modified score	1.51	1.21–1.87	<0.001
Female patient	0.99	0.57–1.71	0.987
Charlson co-morbidity index	1.15	0.76–1.75	0.503
Uses lipid-lowering drugs	0.41	0.23–0.72	0.002

CI, confidence interval; HR, hazard ratio; ICU, intensive care unit.

### 1-Year Follow-up

During 1-year follow-up of idiopathic disease patients, a probable etiology was found only in 15 of these patients (7.5%). Distribution of factors is presented in Table 4.

**Table 4:** Idiopathic AP Patients in Study Given New Etiology Subsequent to 12-month Follow-up.

New Etiology (*n*=15)	Number (%)
Gallstones	4 (26)
Drug-induced	4 (26)
Malignancy	3 (20)
Chronic pancreatitis	3 (20)
Hypertriglyceridemia	1 (8)

## DISCUSSION

In this population-based study of hospitalized AP patients, the proportion with idiopathic disease was above one-third. In comparison to known etiology AP patients, patients with idiopathic disease had milder disease with better prognosis in terms of admission stay, and short-term (30-day) and long-term (1-year) re-admissions. Chronic anti-hypertensive and anti-glycemic medication use was more common among idiopathic AP patients. It suggests that there is a possibility of underestimation of drug-induced pancreatitis.

In our study the most common known cause of AP was gallstones, whereas alcohol consumption was a less prevalent factor. This low prevalence is differ-ent compared to most other countries. Previous studies show a variety of factors causing AP, and the variability is explained by differences in lifestyle and habits. Studies have shown that in most European countries and in the United States there are similar rates of AP caused mainly by alcohol and gallstones.^5^,^6^,^10^,^11^ However, studies carried out in southern European countries such as Greece and Italy show a significant dominance of gallstones as the AP factor.^4^,^12^ In this aspect there is a similarity between the population of southern Israel included in this study and the populations of southern Europe/the Mediterranean basin.

The high percentage of patient admissions for idiopathic AP at SUMC during the period of our study may be a result of undiagnosed gallstones, which is recognized as a possible factor in such cases.^13^,^14^ Additional possibilities are a reporting bias for alcohol consumption and under-diagnosis of drug reactions.^15^

Yet, the percentage of patient admissions for idiopathic AP in our study is similar to findings presented in two large studies carried out within the last 10 years in the United States.^16^,^17^ In another study 36.5% of patients whose AP had no identified factor had a higher risk of death.^18^ That study also showed statistical similarities between groups of patients with idiopathic disease and those with biliary disease as factor (predominantly female and older patients). In another study, patients with alcoholic pancreatitis had the highest risk of dying.^19^

This is the first study to suggest a better prognosis in the subgroup of idiopathic AP, which is mainly expressed in shorter length of hospital stay and lower rates of re-admissions. There was also higher modified Glasgow score and more complications among patients with known etiology compared to patients with idiopathic disease, although this difference was not significant.

As mentioned above, patients with idiopathic disease used more angiotensin receptor blockers (ARBs), angiotensin-converting enzyme (ACE) inhibitors, and drugs for diabetes and hyperlipidemia. This significant difference raises the question as to the presence of a causal association between drug use and incidence of idiopathic AP, especially as we note that idiopathic cases tend to be milder than other cases, similar to drug-induced AP which has been shown to have a better prognosis.^18^ Previous studies have shown that drug-induced AP is a less common form of the disease and is thought to make up only 1%–2% of cases.^19^,^20^

However, the acknowledged difficulty to pinpoint a source in every AP case may mean that drug-induced cases are underdiagnosed. We recommend further research to study the association between usage of the aforementioned medications and incidence of AP, in order to support or refute this hypothesis.

The statistical analysis of the study data was carried out not only to determine clinical and prognostic characteristics of idiopathic AP, but also to try and reveal possible etiologies for the disease. Of the patients with idiopathic AP in our study, 33.3% were followed by a gastroenterology physician, and 12.9% underwent advanced imaging tests during the year following hospitalization, which led to a small proportion (7.5%) of the total number of patients having their disease subsequently reclassified with known etiology.

Previous researchers have proposed that idiopathic cases are actually caused by undiagnosed gallstones and have mentioned various incidence statistics.^14^,^21^ In order to diagnose whether the source of the illness in these cases is indeed gallstones, various methods can be used: testing for increase in liver enzymes, repeating imaging tests, using advanced imaging such as ERCP, MRCP, and EUS, performing *ex vivo* bile crystallization, and cholecystectomy.^7^,^8^ Another common factor in AP is sphincter of Oddi dysfunction, where the assumption is that increased pressure on the sphincter may cause AP.^22^ The preferred method of diagnosis in these cases is ERCP plus manometry.^23^ Additional etiological factors revealed by use of invasive procedures include neoplasia and pancreatic divisum.^24^

In our study, several idiopathic cases could be reclassified as biliary according to high liver enzymes (ALT>150 U/L).^25^ Only four additional cases (2%) could be reclassified as caused by gallstones further to data collected from patients during the year following hospitalization. The small number of patients with idiopathic AP who could be reclassified as having gallstones, along with the differences in clinical and prognostic characteristics between groups, creates doubt as to the assumption that the majority of idiopathic cases are actually due to gallstones.

## STUDY LIMITATIONS

This retrospective study was based on data from a single medical center in Israel. We acknowledge possible underestimation of drug use and alcohol consumption as etiological factors, due to reporting bias. Discharged patients were followed for a relatively short period of 12 months.

## CONCLUSION

In conclusion, idiopathic acute pancreatitis is relatively common among AP patients. According to our study, idiopathic AP seems to be less severe than AP with known etiology. We suggest that a partial explanation for this finding could be the under-diagnosis of drug-induced AP, a milder form of the disease, among patients with no identifying factor. This hypothesis is supported by the increased use of certain drugs in the idiopathic AP group. We recommend that efforts be increased to discover the etiology among idiopathic AP patients. Future research should focus on the use of medication as a possible AP factor.

## Abbreviations

ACE: angiotensin-converting enzyme
AGA: American Gastroenterological Association
ALT: alanine aminotransferase
AP: acute pancreatitis
ARBs: angiotensin receptor blockers
ERCP: endoscopic retrograde cholangiopancreatography
EUS: endoscopic ultrasound
GEE: generalized estimating equation
HR: hazard ratio
ICU: intensive care unit
IQR: interquartile range
MRCP: magnetic resonance cholangiopancreatography
SD: standard deviation
SUMC: Soroka University Medical Center
